# Spectrum of Cytogenetic Abnormalities in Adolescent and Young Adult Patients of B-Cell Acute Lymphoblastic Leukemia in a Tertiary Care Hospital in Pakistan

**DOI:** 10.7759/cureus.83122

**Published:** 2025-04-28

**Authors:** Sana Brohi, Israr A Shaikh, Fatima Tariq, Ammarah Tahir, Imran Iftikhar, Imran A Siddiqui, Syed W Bokhari, Muhammad Tariq Mahmood, Romena Qazi, Usman Ahmad

**Affiliations:** 1 Pathology, Shaukat Khanum Memorial Cancer Hospital and Research Centre, Lahore, PAK; 2 Oncology, Fatima Memorial Hospital, Lahore, PAK; 3 Medical Oncology, Shaukat Khanum Memorial Cancer Hospital and Research Centre, Lahore, PAK; 4 Molecular Biology, Shaukat Khanum Memorial Cancer Hospital and Research Centre, Lahore, PAK

**Keywords:** adolescent, all, b-all, cytogenetic abnormalities, pakistan, philadelphia, young adults

## Abstract

Introduction: Acute lymphocytic leukemia (ALL) is a malignancy of infiltration of B or T precursor lymphoid cells, i.e., lymphoblasts, in bone marrow and other lymphoid organs. Detection of various cytogenetic abnormalities in ALL patients is important for the purpose of prognosis and targeted treatment. The aim of this study is to identify the type and frequency of various cytogenetic abnormalities in adolescent and young adult precursor B-cell ALL (B-ALL) patients in Pakistan and compare them with patients of a similar age group in other European and Asian countries.

Methods: A total of 134 B-ALL patients (112 male, 22 female), aged 18-40 years, were included in this cross-sectional retrospective study, with data from December 1, 2014, to March 30, 2020, conducted in the Department of Clinical Hematology and Oncology at Shaukat Khanum Memorial Cancer Hospital & Research Center, Lahore, Pakistan. Bone marrow samples were received in the laboratory’s cytogenetics section of patients diagnosed as B-ALL on flow cytometry for conventional karyotyping. After proper processing of samples with at least 400-bands level resolution, 20 bone marrow cells were counted and analyzed. Scoring and interpretation of cytogenetics was performed using the International System for Human Cytogenomic Nomenclature (ISCN 2020).

Results: Out of 134 B-ALL patients, 72 (54%) showed cytogenetic abnormalities, while 62 (46%) had normal karyotypes. Of all the B-ALL patients, translocation between chromosomes 9 and 22, t(9;22), was the most commonly detected abnormality (i.e., 13.4%).

Conclusion: Cytogenetic analysis is important for risk stratification and targeted therapy in B-ALL, especially in regions with limited access to advanced molecular diagnostics.

## Introduction

Acute lymphocytic leukemia (ALL) is characterized by genetic alterations that disrupt lymphoid precursor cell differentiation and promote proliferation [1]. These alterations are critical for risk stratification and the development of targeted therapies [2], as well as targeted therapies. WHO Classification of Hematolymphoid Tumors, 5th edition (B-cell lymphoid proliferations and lymphomas) classifies precursor B-cell Neoplasms as a separate entity [3]. Conventional cytogenetics has been used to analyze genome modifications that include genomic gains and losses, as well as rearrangements within and between chromosomes [4]. The detection and assessment of cytogenetic abnormalities play a vital role in prognostication and treatment of this aggressive hematologic malignancy [5].

Most ALL cases exhibit an abnormality either in chromosome number (ploidy) or in their structure (i.e., translocations, deletions, or inversions) [6]. Technical challenges, such as poor chromosome morphology and indistinct banding patterns, often complicate cytogenetic analysis in ALL [5,7]. However, with significant improvements in banding techniques, the rate of detection of chromosomal abnormalities in ALL has gone up to 60-85% [8-12]. According to the Third International Workshop on Chromosomes in Leukemia (TIWCL), only 39% of cytogenetic changes occur in T-cell ALL, the rest being associated with B-cell ALL [8]. 

Secker-Walker et al. first reported the significance of cytogenetics in the prognosis of childhood ALL in 1989 [12]. According to their research, hyperdiploid karyotypes had a better prognosis compared to hypodiploid or pseudodiploid cases. These analytics were further confirmed in the follow-up study [13] as well as by other investigators [10,14,15]. The TIWCL reported that cytogenetic studies can help in the risk stratification of ALL patients, while also giving an insight into their disease-free-survival (DFS), complete remission (CR), and remission duration [8].

Cytogenetic profiling in ALL is crucial for risk stratification and treatment selection, yet limited data exist on adolescents and young adults in Pakistan. Differences in genetic backgrounds may influence the frequency of specific chromosomal abnormalities, impacting prognosis and therapeutic decisions. This study aims to analyze cytogenetic patterns in Pakistani patients and compare them with global data, providing insights into population-specific trends.

## Materials and methods

This was a cross-sectional retrospective study conducted at the Department of Clinical Hematology and Oncology at Shaukat Khanum Memorial Cancer Hospital & Research Center, Lahore, Pakistan. The study was approved by the Institutional Review Board of Shaukat Khanum Memorial Cancer Hospital & Research Center (approval number: EX-13-10-20-20).

A total of 134 patients with B-cell ALL, aged 18-40 years, were recruited from December 1, 2014, to March 30, 2020. Inclusion criteria were patients who presented through the walk-in clinic and were diagnosed with B-ALL via immunophenotyping using flow cytometry at our hospital. Patients within the specified age range were excluded only if cytogenetic analysis was not performed for any reason.

Karyotyping procedure

Our laboratory’s Cytogenetics Section receives samples for the detection of different chromosomal abnormalities by conventional karyotyping from hospital-registered and outside patients diagnosed with B-ALL on immunophenotyping by flow cytometry. Conventional karyotyping is performed on bone marrow samples with ≥20% blast cells by laboratory technologists. Samples are collected in green top sodium/lithium heparin tubes to prevent clotting and maintain sample viability for analysis. The temperature for transport is maintained at 15-25°C, which helps preserve the sample’s integrity until it reaches the laboratory, while ensuring it’s delivered within 24 hours, which is crucial for optimal culture conditions. The bone marrow aspirate sample is then cultured in duplicates/triplicates, depending on the request. Samples are cultured within 48 hours of receipt to optimize metaphase yield from blast cells. Specimens are then stored at 2-8^ o^C after culture and harvested using the hypotonic solution potassium chloride (KCl) at 37°C for swelling the cells, allowing chromosomes to spread out properly for analysis. After that, the slides are prepared using the Hanabi PVI metaphase spreader, a common tool in cytogenetics for ensuring even distribution of the cells. Staining with Dulbecco’s phosphate-buffered saline (PBS) pH 7.0, trypsin, and Harleco Giemsa stain is performed to enhance visibility of the chromosomal structures, ensuring that they are clear and identifiable under the microscope.

Chromosomal analysis

For chromosomal analysis, Cyto-Vision MB8 (Leica Biosystems, Nussloch, Germany), a dedicated software tool for capturing and analyzing cytogenetic data, was used. Neoplastic disorders, like ALL, require the analysis of at least 20 cells (preferably with a minimum of 400 bands level resolution), ensuring that enough data is available to detect any structural or numerical chromosomal abnormalities that may be contributing to the leukemia. Scoring and interpretation of cytogenetics was performed using the International System for Human Cytogenomic Nomenclature (ISCN 2020) [16], as the reference standard, allowing standardization of reports and comparisons with global data. 

## Results

**Figure 1:**
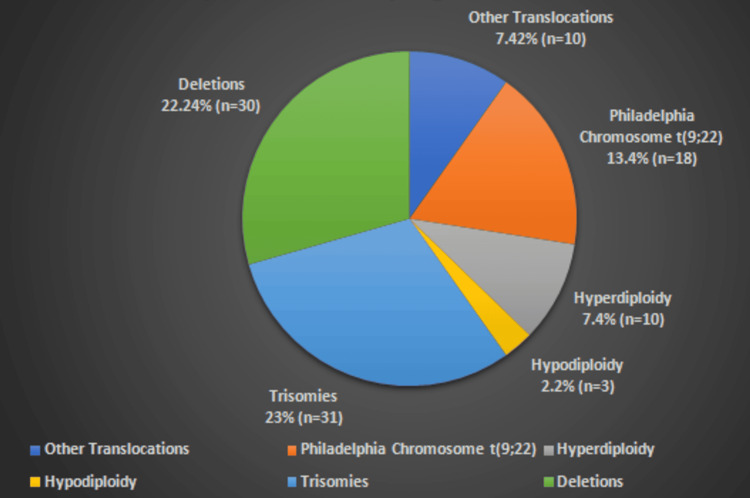
Spectrum of cytogenetic abnormalities in the B-cell acute lymphocytic leukemia patients

Out of 134 patients diagnosed with B-ALL by flow cytometry, 72 (54 %) showed different cytogenetic abnormalities, while 62 (46 %) showed normal karyotype, as shown in Table 1. The summary of all chromosomal abnormalities in B-ALL patients is given in Figure 1.

**Table 1:** Cytogenetic spectrum (N=134)

Cytogenetics	Frequency	Percentage
Abnormal Cytogenetics	72	54%
Normal Cytogenetics	62	46%

Translocations

Of all the screened cytogenetics, translocation between chromosomes 9 and 22, t (9;22), was the commonest abnormality. Translocation between chromosomes 9 and 22, t(9;22), was found in 18 (13.4 %) patients, while translocation between chromosome 1 and 19, t(1;19), was found in five (3.7 %) patients. Other translocations found were t(1;3), t(9;15), t(1;2) and t(5;9), as shown in Table 2.

**Table 2:** Translocations (N=134)

Translocations	Frequency	Percentage
t (9;22)	18	13.4%
t (1;19)	5	3.7%
t (1;3)	2	1.5%
t (9;15)	1	0.74%
t (1;2)	1	0.74%
t (5;9)	1	0.74%

Hyperdiploidy and hypodiploidy

Out of 134 patients analyzed, 10 (7.4%) were hyperdiploid while three (2.2%) showed hypodiploidy, as shown in Table 3.

**Table 3:** Hyperdiploidy and hypodiploidy (N=134)

Hyperdiploidy/Hypodiploidy	Frequency	Percentage
Hyperdiploidy	10	7.4%
Hypodiploidy	3	2.2%

Trisomy

Trisomy 21 (n=5, 3.7%) was the most prevalent anomaly found in this group, followed by trisomy 2 and 12 (n=4, 3%). Other anomalies are shown in Table 4.

**Table 4:** Trisomy (N=134)

Trisomy	Frequency	Percentage
Trisomy 21	5	3.7%
Trisomy 2	4	3%
Trisomy 12	4	3%
Trisomy 1	3	2.2%
Trisomy 5	3	2.2%
Trisomy 7	3	2.2%
Trisomy 4	2	1.5%
Trisomy 8	2	1.5%
Trisomy 3	1	0.74%
Trisomy 6	1	0.74%
Trisomy 9	1	0.74%
Trisomy 10	1	0.74%
Trisomy 13	1	0.74%

Deletions

Deletion 9p was observed in seven (5.2%) patients, followed by deletion 5q, 7p, and 13q, which were found in three (2.2%). Other deletions are shown in Table 5.

**Table 5:** Deletions of short arm (p) and long arms (q) (N=134)

Deletion	Frequency	Percentage
9p	7	5.2%
5q	3	2.2%
7p	3	2.2%
13q	3	2.2%
3q	2	1.5%
11q	2	1.5%
12p	2	1.5%
13p	2	1.5%
3p	1	0.74%
6q	1	0.74%
7q	1	0.74%
8q	1	0.74%
9q	1	0.74%
1p	1	0.74%

Complex karyotype

The WHO defines complex karyotype as the presence of more than or equal to three chromosomal abnormalities [17]. Out of 134 B-ALL patients, 19 (14.1%) showed complex karyotypes.

## Discussion

In our study, 54% (n=72) of patients exhibited chromosomal abnormalities, including structural and/or numerical changes. This aligns closely with findings from another local study (49%) [18], suggesting consistent cytogenetic patterns in Pakistani B-ALL patients. However, Safaei et al. [19] reported the incidence at 61.7% while Lazaryan et al. [20] found chromosomal anomalies in 36.5% of patients.

Translocation between chromosome 9 and 22, i.e., t(9;22), BCR-ABL1, which result in Philadelphia chromosome responsible for activation of a tyrosine kinase, was found in 13.4% patients in our study, which is comparable to frequency of 15% as reported by Moorman et al. [21] and 10% as reported by Shaikh et al. [18]. The second most common translocation found in our study was translocation between chromosomes 1 and 19, t(1;19), with a frequency of 3.7%, resulting in the TCF3:PBX1 protein complex. Yilmaz et al. [22] and Uckun et al. [23] reported t(1;19) in 4% of adults, which closely resembles our finding. As reported by Chen et al., the pediatric population with ALL has a high incidence of hyperdiploidy with a frequency of 17.6%, while adult patients with ALL have a lower frequency of 4.4% [24]. In our study, the frequency of hyperdiploidy in adolescents and young adult B-ALL patients was found to be 7.4%, which is higher than the frequency in adults but lower than the pediatric population as reported in the study by Chen et al. [24], but it is comparable to 6.6% as reported by Shaikh et al. [18]. Hypodiploidy in B-ALL, which carries a poor prognosis, was found in 2.2% of the patients in our study, but it was reported to be 0.6% by Shaikh et al. [18].

The frequency of various trisomies in our study was 3.7%, which is higher than the frequency reported in a study by Raimondi et al. (1.8%) [25]. Abnormalities of the short arm of chromosome 9 are found in approximately 9% of adults [26]. In our study, deletion of the short arm of chromosome 9 (del 9p) was found in 5.2%, and deletion of the long arm of chromosome 5 (i.e., del 5q), which is uncommon in ALL [27], was found in 2.2 %of our study population.

**Figure 2:**
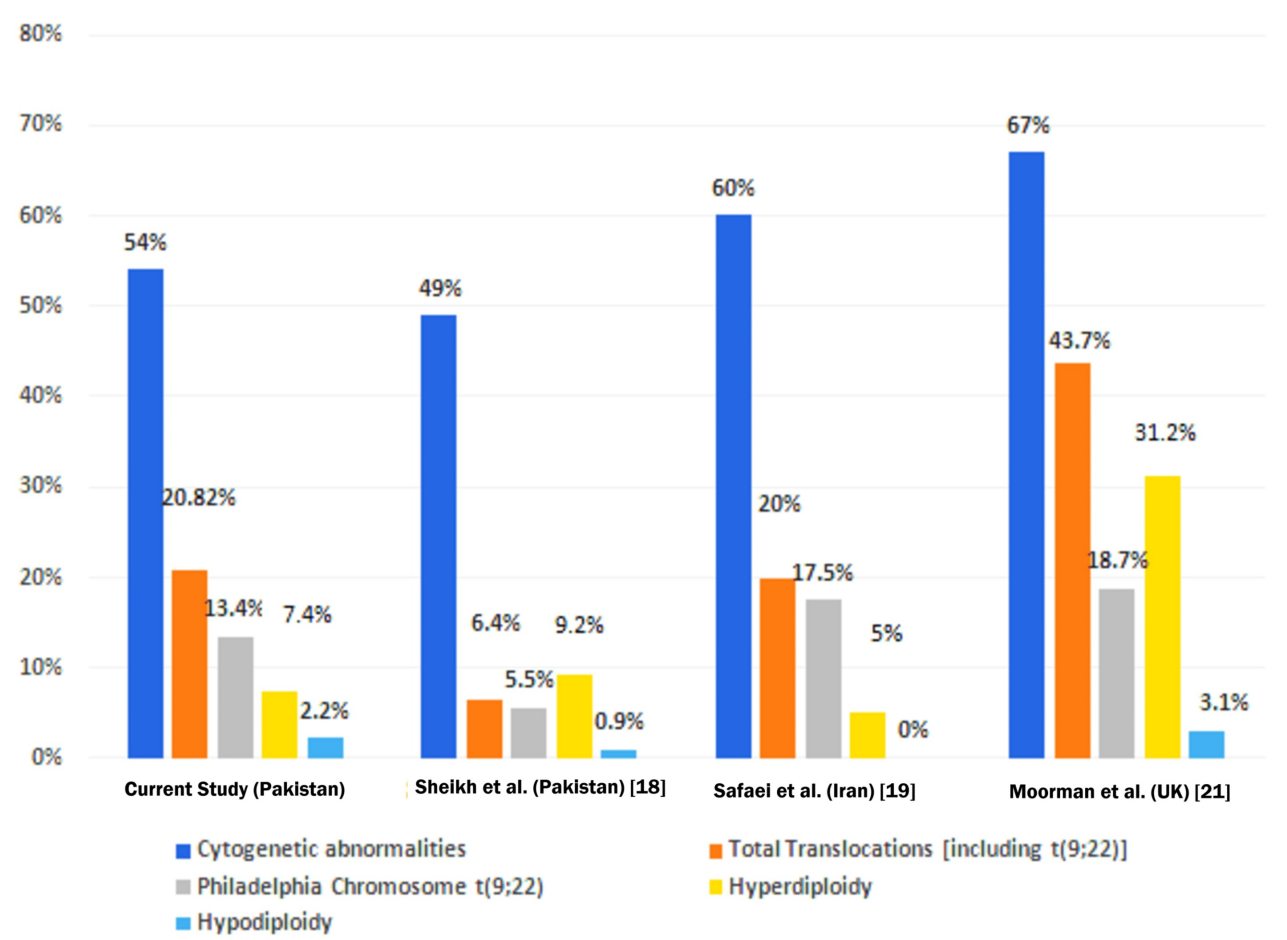
Comparison of cytogenetic abnormalities found in acute lymphocytic leukemia in different populations References: [18,19,21]

Table 6 and Figure 2 show a comparison of cytogenetic abnormalities found in the current study and those found in other studies conducted in Pakistan, Iran, and the United Kingdom in similar age groups. It is to be noted that cytogenetic abnormalities were detected in >50% of the population in each study. Hyperdiploidy is more common than hypodiploidy, and translocations take up to one-third of cytogenetic alterations, with the Philadelphia chromosome t(9; 22) (q34; q11) being the most common abnormality.

**Table 6:** Comparison of cytogenetic abnormalities amongst different studies with adolescent and young adult B-ALL patients B-ALL: B-cell acute lymphocytic leukemia

Parameters	Current study (Pakistan)	Sheikh et al. (Pakistan) [18]	Safaei et al. (Iran) [19]	Moorman et al. (United Kingdom) [21]
Sample number (n)	134	121	46	115
Age (years)	18-40	15-29	35.36±14.82	15-29
Cytogenetic abnormalities (%)	54%	49%	60%	67%
Total translocations Including t(9;22) (%)	20.82%	6.4%	20%	43.7%
Philadelphia Chromosome t(9;22) (%)	13.4%	5.5%	17.5%	18.7%
Hyperdiploidy (%)	7.4%	9.2%	5%	31.2%
Hypodiploidy (%)	2.2%	0.9%	0%	3.1%

Limitations

It is noted that conventional cytogenetics cannot detect cryptic chromosomal abnormalities. Therefore, the finding of 46% of patients with normal karyotypes in our study may harbor undetected abnormalities. Additional molecular techniques, such as fluorescence in situ hybridisation (FISH), polymerase chain reaction (PCR), or next-generation sequencing (NGS), are required to fully exclude genetic alterations in these cases.

## Conclusions

Our findings highlight the importance of cytogenetic analysis in B-ALL for risk stratification and targeted therapy, particularly in regions with limited access to advanced molecular diagnostics. However, further research is warranted to assess genetic alterations in B-ALL patients using advanced methodologies such as FISH, PCR, or NGS, which could reveal novel mutations and help refine personalized treatment approaches.
